# Supplementary material for: Endozoicomonas dominance and Vibrionaceae stability underpin resilience in urban coral Madracis auretenra
Source: PeerJ. 2025 Apr 15;13:e19226. doi: 10.7717/peerj.19226 (PMC12007501; doi:10.7717/peerj.19226)
Supplement: Supplemental Information 1 [file peerj-13-19226-s001.docx]

**Supplementary Figures**

| **A** | 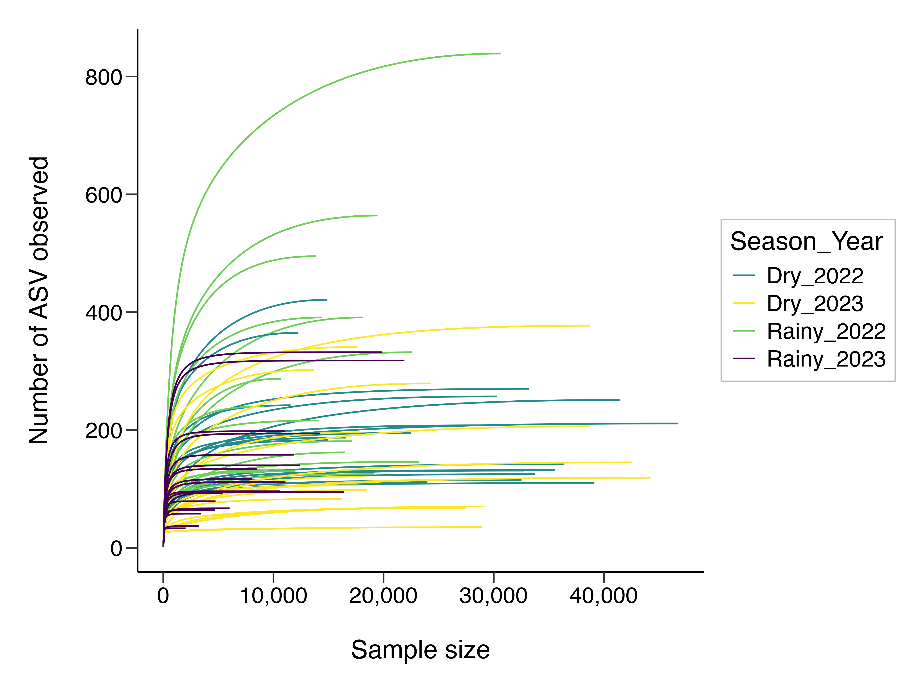 | **B** | 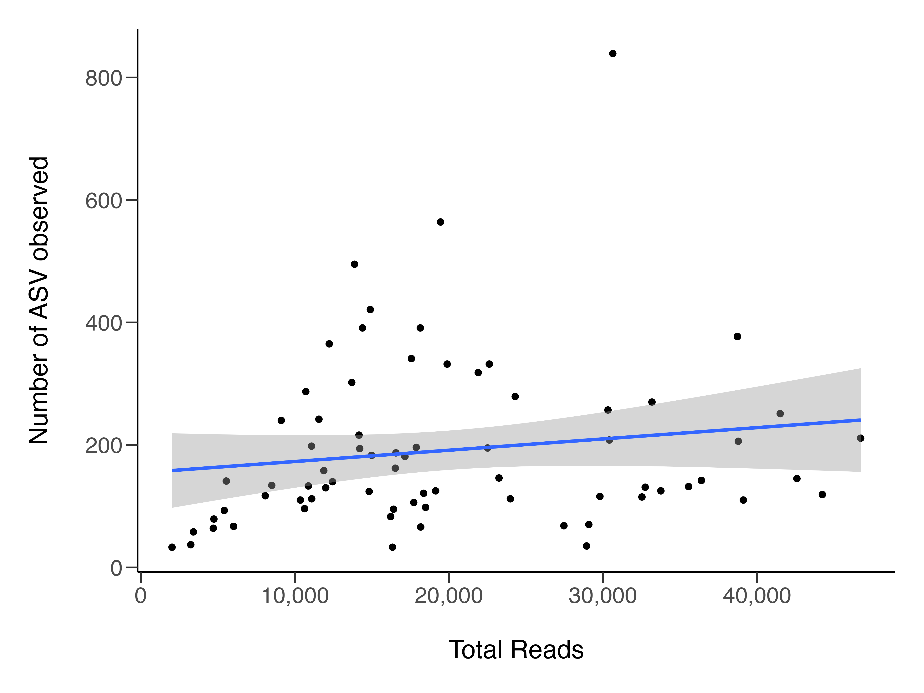 |
| --- | --- | --- | --- |

**Figure S1. Rarefaction curves, and correlation between ASVs and total reads.** **(A)** Curves show the number of observed ASV related to the sequencing depth (sample size). A steep incline and early plateau are reached for all samples indicating enough sequencing effort due to few new sequences being detected with increasing sequencing sample size. **(B)** The correlation between total reads and the observed ASV shows the increase in richness as the number of reads increases as expected.

**
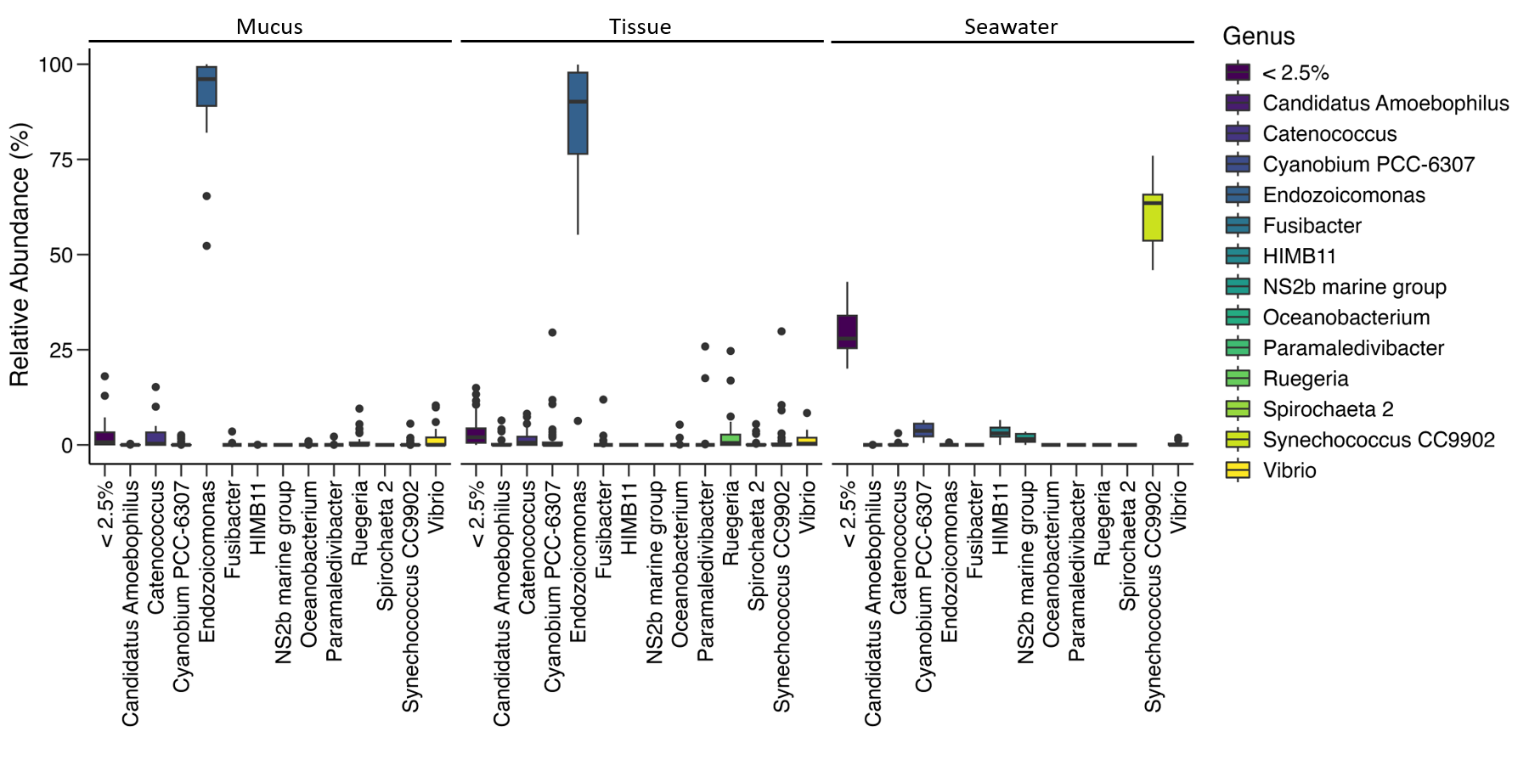
****Figure S2. Microbiome composition of coral *Madracis auretenra* (mucus and tissue) and seawater at the genus level.** The mean relative abundance (%) of the thirteen most abundant genera is plotted, and the remaining taxa are grouped under abundance <2.5%.

| 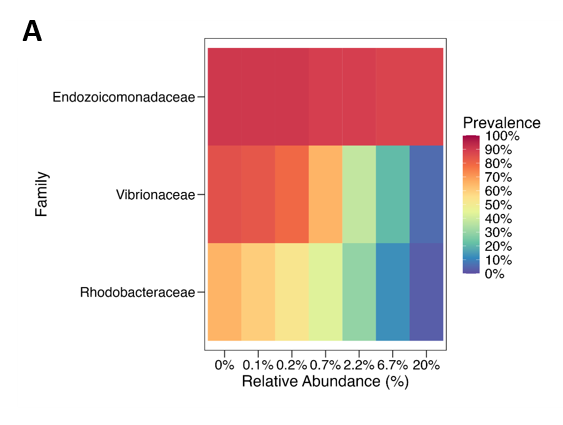 |
| --- |
| 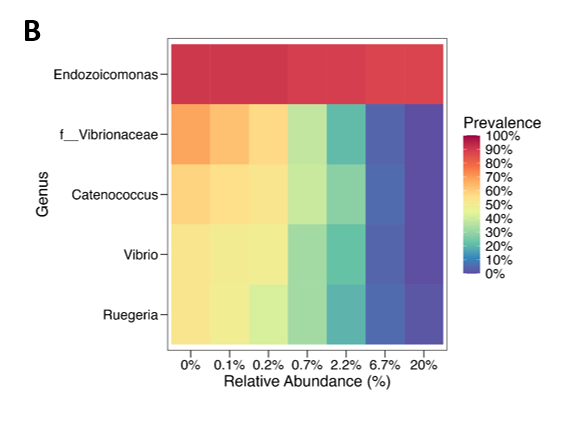 |

**Figure S3. The core microbiome of coral Madracis auretenra.** Heatmap of **(A)** core bacterial families and **(B)** genera representing the taxa with a prevalence >50% and a relative abundance >0.2%. Cells are scaled by the prevalence (%). The core microbiome of coral M. auretenra is represented by Endozoicomonas (prevalence >90%), Catenococcus (prevalence >70%), Vibrio (prevalence >60%), and Ruegeria (prevalence >50%).


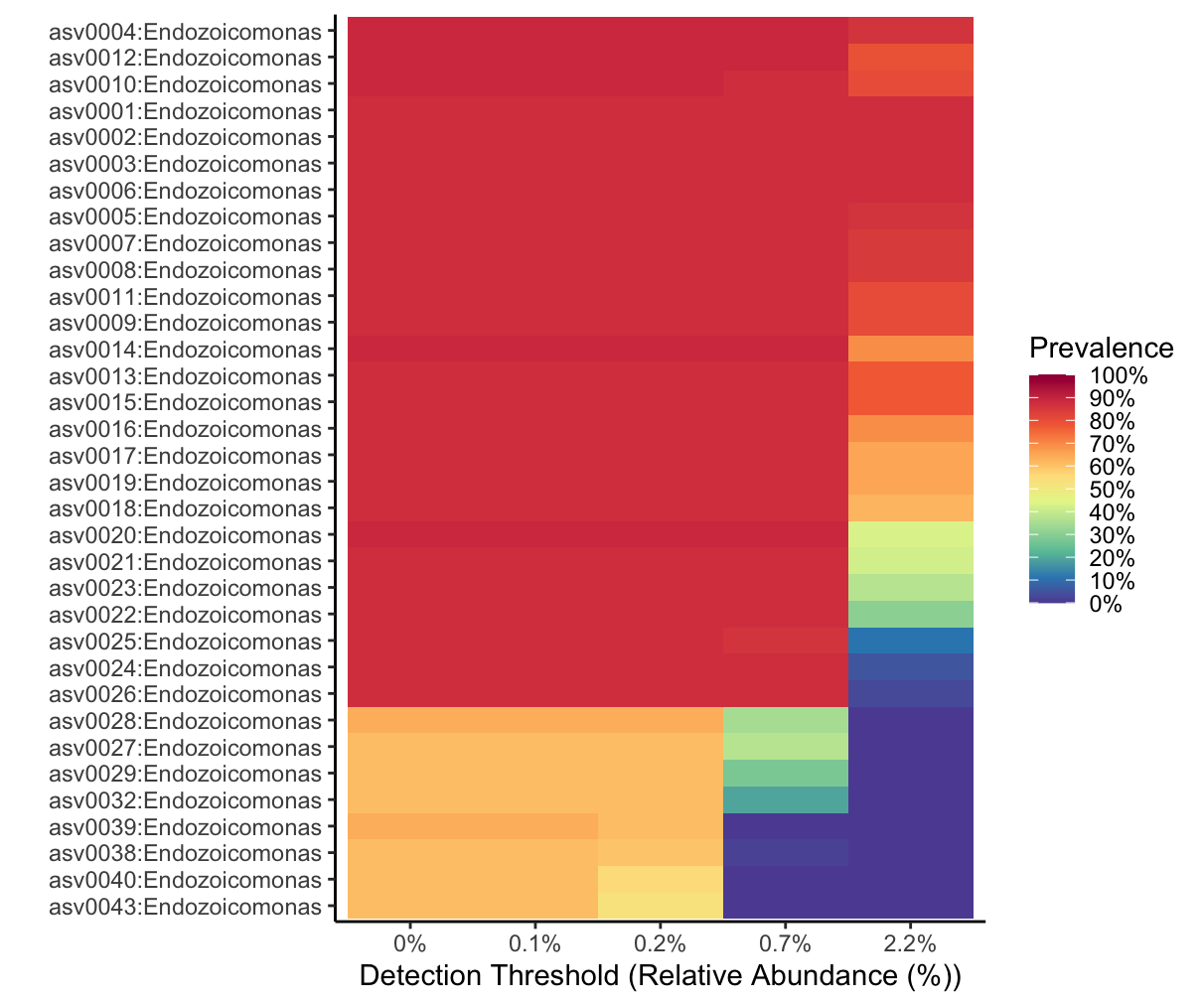


**Figure S4. ASVs conforming the core microbiome of coral *Madracis auretenra*.** Heatmap showing the most prevalent and abundant ASVs of the core microbiome. Asv0004, asv0012, asv0010, asv0001-3, and asv0006 belonging to the genus Endozoicomonas were identified as the most prevalent taxa dominating the core microbiome with a prevalence of ~90%.

**
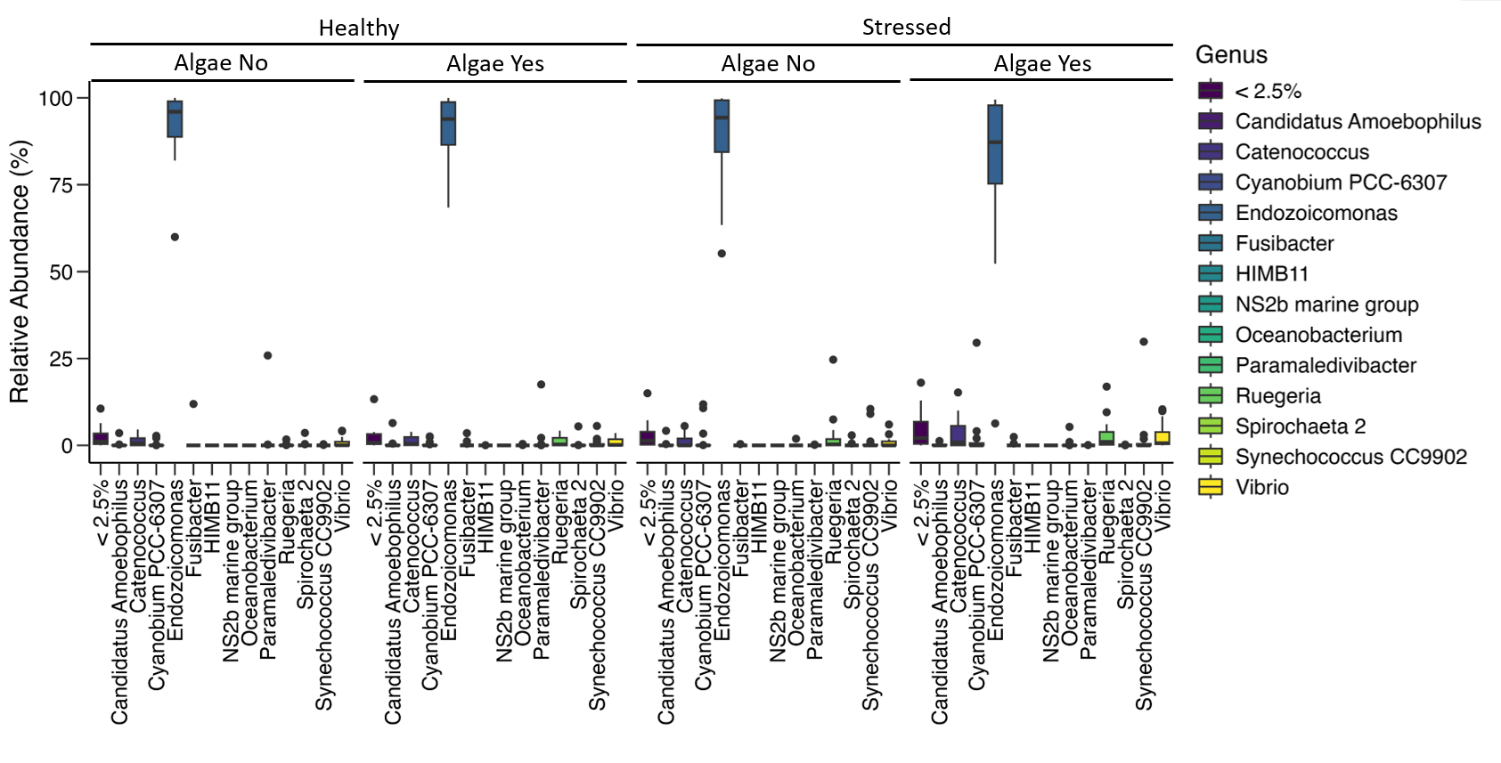
**

**Figure S5. Microbiome composition of coral Madracis auretenra according to the health status and contact with algae conditions.** The mean relative abundance (%) of the thirteen most abundant genera is plotted by grouping by coral health status (healthy and stressed) and contact with algae (Yes and No). The remaining taxa are grouped under abundance <2.5%.


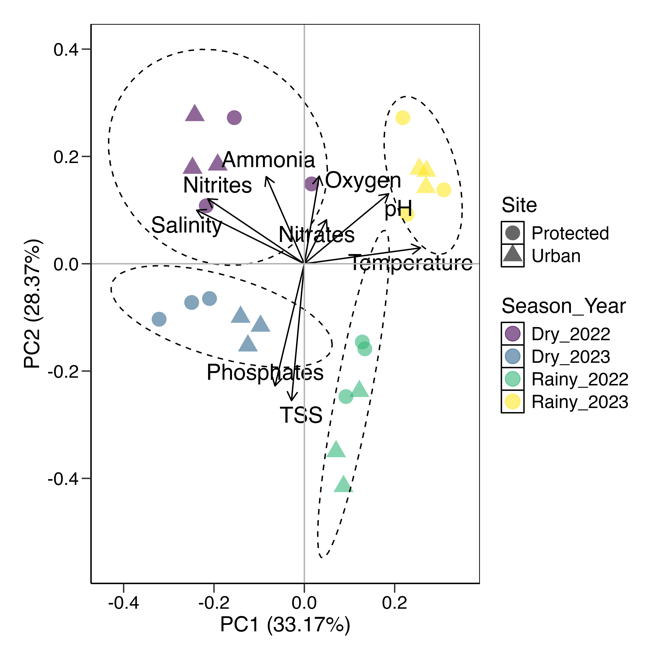


**Figure S6. Principal Component Analysis (PCA).** Physicochemical variables are summarized in two components: PC1 (proportion=33.17%) and PC2 (proportion=28.37%), which explained more than 60% of the variation of the physicochemical variables among seasons (PERMANOVA; pseudo-F(_3, 23)_ =25.18, p=0.001, perms=998). PC1 summarized temperature, pH, nitrites, and salinity, whereas PC2 summarized oxygen saturation, ammonia, nitrites, orthophosphates, and TSS. Perms=permutations, TSS=total suspended solids.


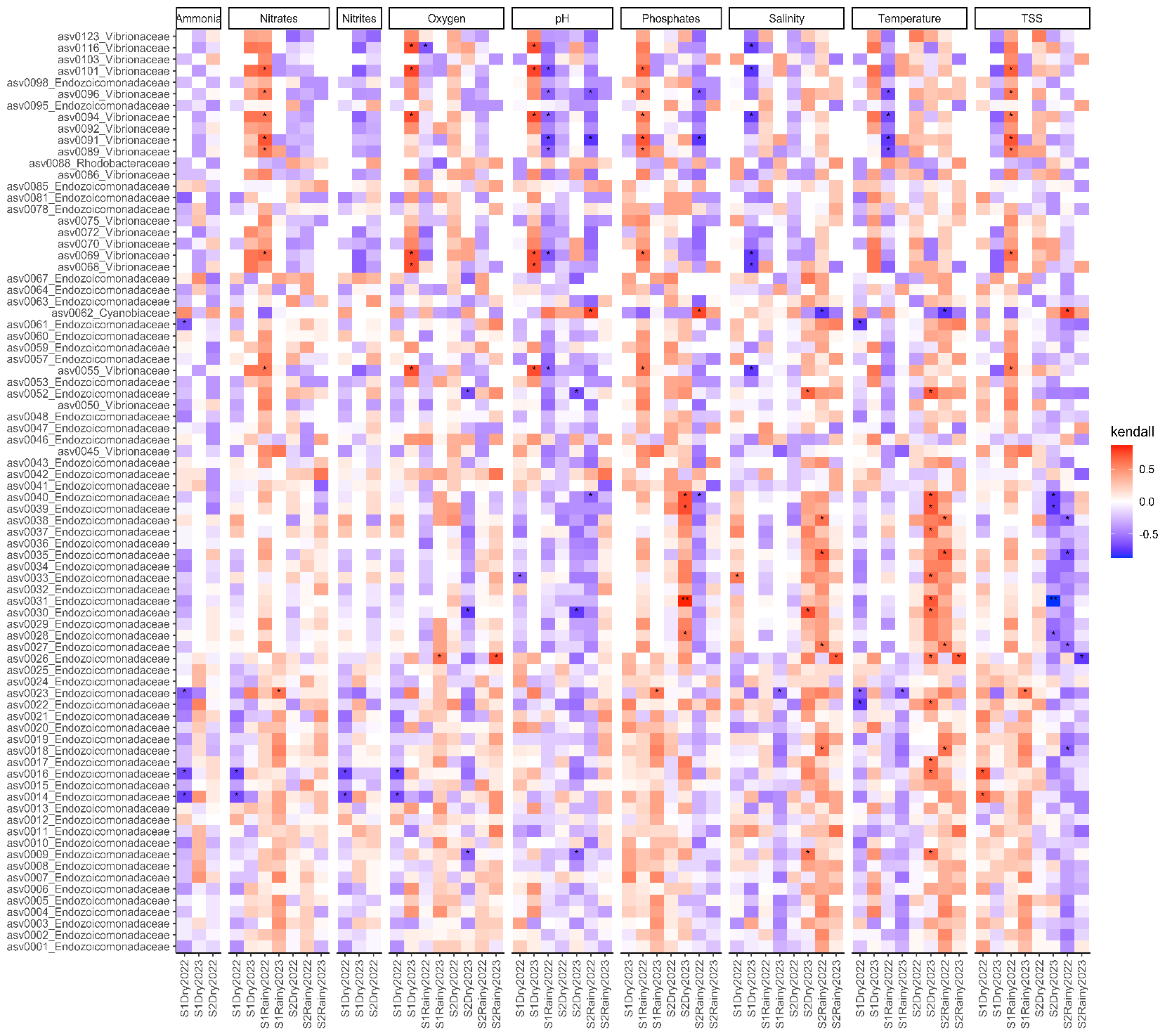


**Figure S7.** **Correlations between the environmental variables and the relative abundance of the most abundant taxa.** Heatmap of the Kendall rank correlation coefficient analysis. Axis X corresponds to samples and axis Y to taxa (ASVs). Significance was detected with adjusted p-values less than 0.05 (*) and less than 0.01 (**). Red color indicates strong positive correlation whereas blue color indicates strong negative correlation.


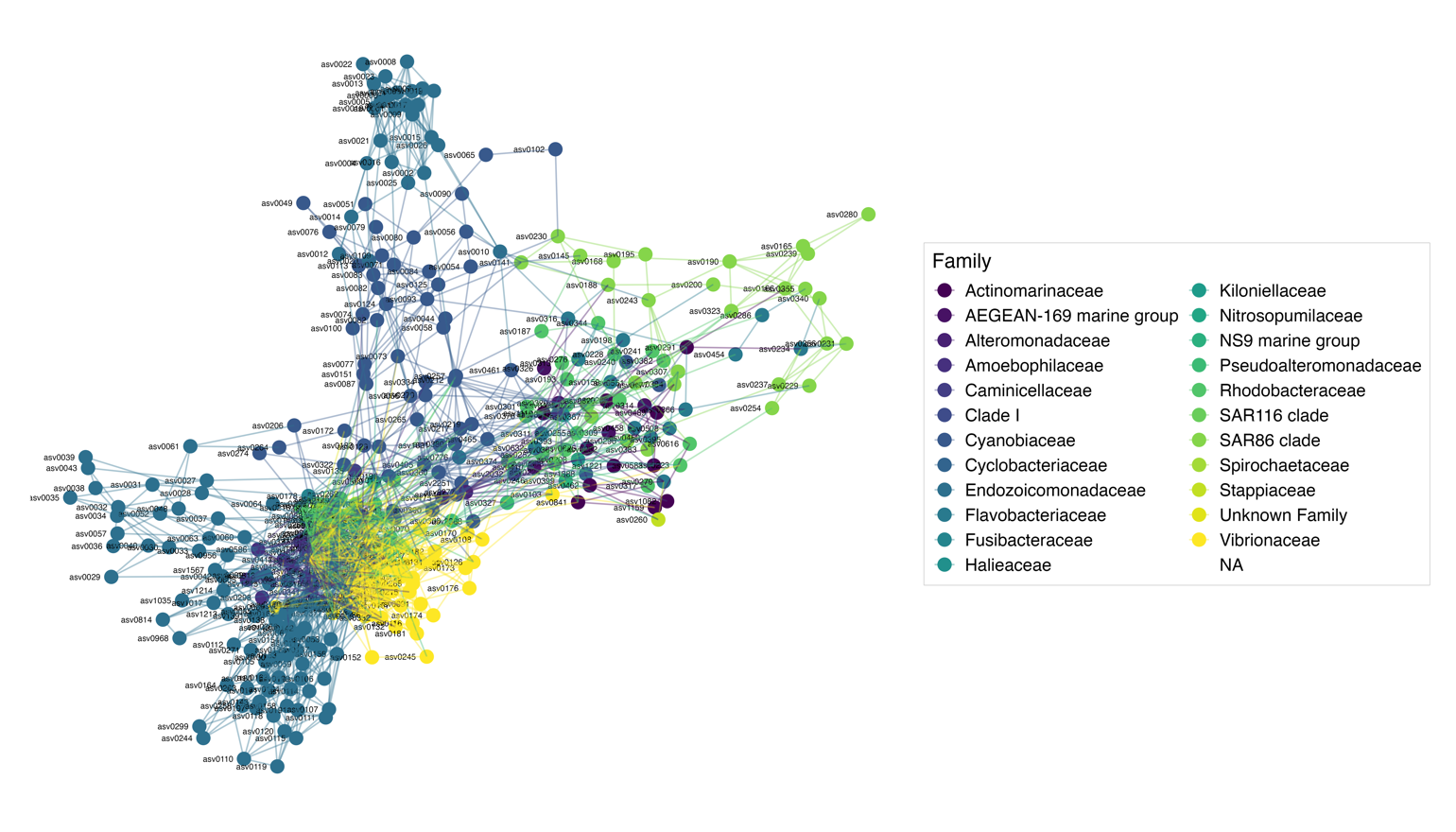


**Figure S8. Co-occurrence network showing the inferred interactions between the different taxa.** The figure represents a microbial interaction network where each node is an ASV, and each edge represents a statistically inferred interaction. The nodes are color-coded based on the taxonomic family). The network topology suggests a mixture of tightly interconnected clusters and more peripheral nodes. Different clusters suggest microbial functional guilds responding to specific coral niches (e.g., mucus layer, skeleton, tissue). Highly connected nodes could be critical players in microbiome resilience. A highly interconnected core (yellow + blue) suggests dominant taxa in the coral microbiome. Peripheral clusters (green, purple) indicate niche-specific or transient microbial groups. Negative interactions suggest competition between opportunistic/pathogenic taxa and beneficial coral-associated bacteria.
