# Supplementary material for: Endozoicomonas dominance and Vibrionaceae stability underpin resilience in urban coral Madracis auretenra
Source: PeerJ. 2025 Apr 15;13:e19226. doi: 10.7717/peerj.19226 (PMC12007501; doi:10.7717/peerj.19226)
Supplement: Supplemental Information 2 [file peerj-13-19226-s002.docx]

**Supplementary Tables**

**Table S1. DNA properties and total reads after sequencing.** The sample size (n), and mean ± standard error (SE) of total DNA amount (µg), DNA purity, input reads and total reads after filtering steps are shown.

| **Compartment** | **n** | **Total DNA**  **(µg)** | **DNA purity**  **(260/280)** | **Input reads** | **Total reads*** |
| --- | --- | --- | --- | --- | --- |
| Healthy mucus | 16 | 2.37 ± 0.44 | 1.75 ± 0.08 | 35,245.50 ± 3,189.10 | 19,716.00 ± 2,897.64 |
| Stressed mucus | 16 | 3.20 ± 0.54 | 1.97 ± 0.09 | 35,332.13 ± 3,888.48 | 19,167.06 ± 2,677.66 |
| Healthy tissue | 16 | 2.31 ± 0.23 | 1.77 ± 0.09 | 39,417.94 ± 3,767.11 | 21,778.69 ± 3,241.57 |
| Stressed tissue | 16 | 3.28 ± 0.79 | 1.86 ± 0.09 | 37,146.25 ± 3,086.09 | 19,386.19 ± 3,007.92 |
| Seawater | 8 | 2.05 ± 0.54 | 2.27 ± 0.14 | 43,656.25 ± 5,706.72 | 18,178.38 ± 2,136.12 |

*After filtering, denoising, merging, and removing mitochondria and chloroplasts.

**Table S2. Statistical analysis of alpha diversity measures.** P-values from the Kruskal-Wallis test of the observed richness and Shannon index diversity measures, and adjusted p-values from pair-wise comparisons. Df=degrees of freedom.

| **Factor** | **Comparison** | **Observed richness** | | | | **Shannon diversity** | | | |
| --- | --- | --- | --- | --- | --- | --- | --- | --- | --- |
|  |  | ***Chi^2^*** | **df** | ***p-*value** | ***p-*adjusted** | ***Chi^2^*** | **df** | ***p-*value** | **Adjusted-*p*** |
| **Site** | Protected vs Urban | 6.94 | 1 | 0.008** | 0.005** | 12.35 | 1 | 0.000** | 0.000** |
| **Season** | Dry 2022 vs Dry 2023 | 31.67 | 3 | 0.000** | 0.000** | 22.98 | 3 | 0.000** | 0.000** |
|  | Rainy 2022 vs Dry 2022 |  |  |  | 0.625 |  |  |  | 0.520 |
|  | Rainy 2023 vs Dry 2022 |  |  |  | 0.000** |  |  |  | 0.019** |
|  | Rainy 2022 vs Dry 2023 |  |  |  | 0.001** |  |  |  | 0.000** |
|  | Rainy 2023 vs Dry 2023 |  |  |  | 0.993 |  |  |  | 0.320 |
|  | Rainy 2023 vs Rainy 2022 |  |  |  | 0.000** |  |  |  | 0.000** |
| **Contact with algae** | “In contact” vs “non-contact” | 0.45 | 1 | 0.497 | 0.545 | 1.01 | 1 | 0.313 | 0.249 |
| **Health status** | Healthy vs Stressed | 0.27 | 1 | 0.605 | 0.472 | 1.49 | 1 | 0.221 | 0.225 |
| **Compartment** | Seawater vs Mucus | 5.32 | 2 | 0.069 | 0.053 | 13.63 | 2 | 0.001** | 0.000** |
|  | Tissue vs Mucus |  |  |  | 0.999 |  |  |  | 0.690 |
|  | Tissue vs Seawater |  |  |  | 0.055 |  |  |  | 0.000** |

**Significant difference (*p*<0.05)

**Table S3. Alpha diversity measures.** Mean ± standard error (SE) for alpha diversity measures of the microbial communities of coral *Madracis auretenra* after pooling by factor and level. Observed richness, Shannon index, Gini-Simpson, and Chao1 are shown.

| **Factor** | **Level** | **Observed** | | | **Shannon** | | | **Gini-Simpson** | | | **Chao1** | | |
| --- | --- | --- | --- | --- | --- | --- | --- | --- | --- | --- | --- | --- | --- |
| **Site (Location)** | Protected | 73.86 | ± | 5.63 | 3.67 | ± | 0.06 | 0.97 | ± | 0.001 | 74.28 | ± | 5.66 |
|  | Urban | 90.97 | ± | 5.55 | 3.95 | ± | 0.05 | 0.97 | ± | 0.001 | 91.09 | ± | 5.57 |
| **Season (by year)** | Dry-2022 | 113.28 | ± | 5.46 | 3.98 | ± | 0.08 | 0.97 | ± | 0.002 | 113.56 | ± | 5.48 |
|  | Rainy-2022 | 95.22 | ± | 5.70 | 4.02 | ± | 0.05 | 0.98 | ± | 0.001 | 95.44 | ± | 5.25 |
|  | Dry-2023 | 65.72 | ± | 5.52 | 3.56 | ± | 0.07 | 0.97 | ± | 0.002 | 66.31 | ± | 5.60 |
|  | Rainy-2023 | 55.44 | ± | 7.55 | 3.69 | ± | 0.09 | 0.97 | ± | 0.002 | 55.44 | ± | 7.55 |
| **Contact with algae** | No | 77.53 | ± | 6.34 | 3.71 | ± | 0.05 | 0.97 | ± | 0.001 | 77.82 | ± | 6.37 |
|  | Yes | 81.94 | ± | 5.74 | 3.80 | ± | 0.06 | 0.97 | ± | 0.001 | 82.25 | ± | 5.75 |
| **Health status** | Healthy | 78.31 | ± | 5.48 | 3.70 | ± | 0.05 | 0.97 | ± | 0.001 | 78.68 | ± | 5.51 |
|  | Stressed | 81.16 | ± | 6.58 | 3.80 | ± | 0.06 | 0.97 | ± | 0.001 | 81.38 | ± | 6.60 |
| **Compartment** | Mucus | 78.22 | ± | 6.53 | 3.73 | ± | 0.06 | 0.97 | ± | 0.001 | 78.30 | ± | 6.54 |
|  | Tissue | 81.25 | ± | 5.54 | 3.77 | ± | 0.06 | 0.97 | ± | 0.001 | 81.76 | ± | 5.57 |
|  | Seawater | 103.88 | ± | 11.18 | 4.29 | ± | 0.09 | 0.98 | ± | 0.001 | 103.94 | ± | 11.21 |

**Table S4. Statistical analysis of beta diversity.** P-values from the PERMANOVA (adonis test) analysis using Bray-Curtis dissimilarity, and adjusted p-values from pair-wise comparisons after Benjamin-Hochberg (BH) corrections. Factors: Site (Protected and Urban levels), Season (Dry-2022, Rainy-2022, Dry-2023, and Rainy-2023), and Source (Mucus, Tissue, and Seawater). Df=degrees of freedom.

| **Factor** | **Comparison** | **df** | ***F* model** | **R^2^** | ***p-*value** | **Adjusted-*p*** |
| --- | --- | --- | --- | --- | --- | --- |
| **Site** | Protected vs Urban | 1 | 2.31 | 0.032 | 0.004** | 0.167 |
| **Season** | Dry 2022 vs Dry 2023 | 3 | 2.53 | 0.100 | 0.022** | 0.252  0.268  0.252  0.252  0.573  0.268 |
|  | Rainy 2022 vs Dry 2022 |  |  |  |  |  |
|  | Rainy 2023 vs Dry 2022 |  |  |  |  |  |
|  | Rainy 2022 vs Dry 2023 |  |  |  |  |  |
|  | Rainy 2023 vs Dry 2023 |  |  |  |  |  |
|  | Rainy 2023 vs Rainy 2022 |  |  |  |  |  |
| **Source** | Mucus vs Tissue | 2 | 48.93 | 0.586 | 0.001** | 0.074 |
|  | Mucus vs Seawater |  |  |  |  | 0.001** |
|  | Tissue vs Seawater |  |  |  |  | 0.001** |

**Significant difference (*p*<0.05)

**Table S5. Relative abundance at the family level.** Mean ± standard error (SE) for relative abundance of the families at the microbial communities of coral *Madracis auretenra* after pooling by compartments.

|  | **Compartment** | | | | | | | | |  |
| --- | --- | --- | --- | --- | --- | --- | --- | --- | --- | --- |
| **Family** | **Mucus** | | | **Tissue** | | | **Seawater** | | |  |
| < 2.5% | 0.46 | ± | 0.16 | 1.10 | ± | 0.32 | 8.67 | ± | 1.44 | |
| Amoebophilaceae | 0.04 | ± | 0.01 | 0.57 | ± | 0.25 | 0.00 | ± | 0.00 | |
| Caminicellaceae | 0.07 | ± | 0.07 | 1.39 | ± | 0.96 | 0.00 | ± | 0.00 | |
| Cyanobiaceae | 0.56 | ± | 0.28 | 3.89 | ± | 2.01 | 65.37 | ± | 3.84 | |
| Endozoicomonadaceae | 92.09 | ± | 1.86 | 84.20 | ± | 3.41 | 0.08 | ± | 0.08 | |
| Flavobacteriaceae | 0.00 | ± | 0.00 | 0.00 | ± | 0.00 | 4.94 | ± | 0.82 | |
| Fusibacteraceae | 0.14 | ± | 0.11 | 0.50 | ± | 0.38 | 0.00 | ± | 0.00 | |
| Rhodobacteraceae | 1.06 | ± | 0.38 | 2.81 | ± | 1.06 | 7.11 | ± | 1.27 | |
| SAR86 clade | 0.00 | ± | 0.00 | 0.00 | ± | 0.00 | 12.54 | ± | 1.80 | |
| Spirochaetaceae | 0.00 | ± | 0.00 | 0.43 | ± | 0.21 | 0.00 | ± | 0.00 | |
| Unknown Family | 0.00 | ± | 0.00 | 0.76 | ± | 0.38 | 0.00 | ± | 0.00 | |
| Vibrionaceae | 5.58 | ± | 1.63 | 4.35 | ± | 1.07 | 1.28 | ± | 0.66 | |

**Table S6. Relative abundance (%) of microbial families at the protected location.** Mean ± standard error (SE) of the relative abundance by season and compartment.

| **Family** | **Protected** | | | | | | | | | | | | | | | | | |
| --- | --- | --- | --- | --- | --- | --- | --- | --- | --- | --- | --- | --- | --- | --- | --- | --- | --- | --- |
|  | **Dry** | | | | | | | | | **Rainy** | | | | | | | | |
|  | **Mucus** | | | **Tissue** | | | **Water** | | | **Mucus** | | | **Tissue** | | | **Water** | | |
| < 2.5% | 0.00 | ± | 0.00 | 0.12 | ± | 0.05 | 7.97 | ± | 0.08 | 1.24 | ± | 0.52 | 3.14 | ± | 0.91 | 13.24 | ± | 3.98 |
| Amoebophilaceae | 0.02 | ± | 0.01 | 0.11 | ± | 0.06 | 0.00 | ± | 0.00 | 0.10 | ± | 0.04 | 1.98 | ± | 0.87 | 0.01 | ± | 0.01 |
| Caminicellaceae | 0.00 | ± | 0.00 | 0.03 | ± | 0.03 | 0.00 | ± | 0.00 | 0.00 | ± | 0.00 | 3.23 | ± | 3.23 | 0.00 | ± | 0.00 |
| Cyanobiaceae | 0.00 | ± | 0.00 | 0.01 | ± | 0.01 | 57.70 | ± | 10.98 | 0.11 | ± | 0.11 | 10.45 | ± | 7.44 | 62.05 | ± | 7.62 |
| Endozoicomonadaceae | 99.65 | ± | 0.12 | 98.74 | ± | 0.28 | 0.32 | ± | 0.32 | 84.83 | ± | 4.99 | 64.57 | ± | 9.38 | 0.00 | ± | 0.00 |
| Flavobacteriaceae | 0.00 | ± | 0.00 | 0.00 | ± | 0.00 | 6.79 | ± | 1.14 | 0.00 | ± | 0.00 | 0.00 | ± | 0.00 | 3.85 | ± | 0.43 |
| Fusibacteraceae | 0.00 | ± | 0.00 | 0.00 | ± | 0.00 | 0.00 | ± | 0.00 | 0.44 | ± | 0.44 | 0.35 | ± | 0.30 | 0.00 | ± | 0.00 |
| Rhodobacteraceae | 0.04 | ± | 0.02 | 0.07 | ± | 0.03 | 11.91 | ± | 1.67 | 2.08 | ± | 1.21 | 7.84 | ± | 3.72 | 7.11 | ± | 0.15 |
| SAR86 clade | 0.00 | ± | 0.00 | 0.00 | ± | 0.00 | 14.36 | ± | 8.88 | 0.00 | ± | 0.00 | 0.00 | ± | 0.00 | 10.95 | ± | 0.58 |
| Spirochaetaceae | 0.00 | ± | 0.00 | 0.06 | ± | 0.05 | 0.00 | ± | 0.00 | 0.00 | ± | 0.00 | 1.53 | ± | 0.76 | 0.00 | ± | 0.00 |
| Unknown Family | 0.00 | ± | 0.00 | 0.06 | ± | 0.05 | 0.00 | ± | 0.00 | 0.00 | ± | 0.00 | 2.71 | ± | 1.33 | 0.00 | ± | 0.00 |
| Vibrionaceae | 0.29 | ± | 0.10 | 0.79 | ± | 0.24 | 0.95 | ± | 0.95 | 11.20 | ± | 4.50 | 4.20 | ± | 1.10 | 2.79 | ± | 2.79 |

**Table S7. Relative abundance (%) of microbial families at the urban location.** Mean ± standard error (SE) of the relative abundance by season and compartment.

| **Family** | **Urban** | | | | | | | | | | | | | | | | | |
| --- | --- | --- | --- | --- | --- | --- | --- | --- | --- | --- | --- | --- | --- | --- | --- | --- | --- | --- |
|  | **Dry** | | | | | | | | | **Rainy** | | | | | | | | |
|  | **Mucus** | | | **Tissue** | | | **Water** | | | **Mucus** | | | **Tissue** | | | **Water** | | |
| < 2.5% | 0.07 | ± | 0.07 | 0.14 | ± | 0.09 | 5.20 | ± | 0.08 | 0.53 | ± | 0.22 | 1.01 | ± | 0.91 | 8.29 | ± | 3.98 |
| Amoebophilaceae | 0.02 | ± | 0.02 | 0.13 | ± | 0.07 | 0.00 | ± | 0.00 | 0.00 | ± | 0.00 | 0.08 | ± | 0.87 | 0.00 | ± | 0.01 |
| Caminicellaceae | 0.00 | ± | 0.00 | 0.06 | ± | 0.04 | 0.00 | ± | 0.00 | 0.29 | ± | 0.27 | 2.22 | ± | 3.23 | 0.00 | ± | 0.00 |
| Cyanobiaceae | 0.03 | ± | 0.02 | 0.10 | ± | 0.06 | 76.52 | ± | 10.98 | 2.09 | ± | 0.96 | 5.00 | ± | 7.44 | 65.22 | ± | 7.62 |
| Endozoicomonadaceae | 95.96 | ± | 1.70 | 92.54 | ± | 2.73 | 0.00 | ± | 0.32 | 87.91 | ± | 3.64 | 80.93 | ± | 9.38 | 0.00 | ± | 0.00 |
| Flavobacteriaceae | 0.00 | ± | 0.00 | 0.00 | ± | 0.00 | 2.56 | ± | 1.14 | 0.02 | ± | 0.01 | 0.00 | ± | 0.00 | 6.58 | ± | 0.43 |
| Fusibacteraceae | 0.00 | ± | 0.00 | 0.00 | ± | 0.00 | 0.00 | ± | 0.00 | 0.12 | ± | 0.08 | 1.63 | ± | 0.30 | 0.00 | ± | 0.00 |
| Rhodobacteraceae | 0.36 | ± | 0.20 | 0.78 | ± | 0.25 | 3.51 | ± | 1.67 | 1.75 | ± | 0.83 | 2.55 | ± | 3.72 | 5.92 | ± | 0.15 |
| SAR86 clade | 0.00 | ± | 0.00 | 0.00 | ± | 0.00 | 11.65 | ± | 8.88 | 0.00 | ± | 0.00 | 0.00 | ± | 0.00 | 13.18 | ± | 0.58 |
| Spirochaetaceae | 0.00 | ± | 0.00 | 0.02 | ± | 0.02 | 0.00 | ± | 0.00 | 0.00 | ± | 0.00 | 0.10 | ± | 0.76 | 0.00 | ± | 0.00 |
| Unknown Family | 0.00 | ± | 0.00 | 0.00 | ± | 0.00 | 0.00 | ± | 0.00 | 0.00 | ± | 0.00 | 0.28 | ± | 1.33 | 0.00 | ± | 0.00 |
| Vibrionaceae | 3.56 | ± | 1.71 | 6.22 | ± | 2.75 | 0.56 | ± | 0.95 | 7.29 | ± | 3.81 | 6.20 | ± | 1.10 | 0.81 | ± | 2.79 |

**Table S8. Analysis of the Composition of Microbiomes with Bias Correction (ANCOM-BC).** Families were identified as differentially abundant with p-values less than 0.05 using negative binomial generalized linear models (GLM). The thirteen most representative families, Log fold changes and p-values are shown.

| **Taxon** | **Source**  **(Mucus-Tissue)** | | **Site** | | **Season** | | **Algae contact** | | **Health status** | |
| --- | --- | --- | --- | --- | --- | --- | --- | --- | --- | --- |
|  | Log Fold | *p*-value | Log Fold | *p*-value | Log Fold | *p*-value | Log Fold | *p*-value | Log Fold | *p*-value |
| Endozoicomonadaceae | –1.40 | 0.00** | –0.10 | 0.72 | –2.17 | 0.00** | –0.31 | 0.27 | –0.08 | 0.77 |
| Vibrionaceae | –0.87 | 0.38 | 0.40 | 0.53 | –0.88 | 0.19 | 0.52 | 0.41 | 0.59 | 0.35 |
| Rhodobacteraceae | –0.00 | 1.00 | 0.96 | 0.06 | 0.43 | 0.44 | 1.66 | 0.00** | 2.08 | 0.00** |
| Pseudoalteromonadaceae | –1.26 | 0.01** | –0.81 | 0.09 | 1.41 | 0.01** | 0.05 | 0.91 | –0.32 | 0.49 |
| Fusibacteraceae | –0.92 | 0.00** | –0.18 | 0.66 | 0.31 | 0.49 | 0.13 | 0.76 | –0.05 | 0.91 |
| Cyanobiaceae | –0.14 | 0.79 | 1.46 | 0.01** | 1.51 | 0.01** | 0.63 | 0.23 | 0.64 | 0.22 |
| Spirochaetaceae | 0.25 | 0.00** | –0.82 | 0.03** | –0.34 | 0.41 | –0.23 | 0.54 | –0.24 | 0.52 |
| Caminicellaceae | –0.45 | 0.00** | 0.21 | 0.64 | –0.17 | 0.73 | –0.14 | 0.76 | –0.36 | 0.42 |
| Amoebophilaceae | 0.13 | 0.79 | –1.02 | 0.04** | –0.78 | 0.14 | 0.02 | 0.96 | –0.05 | 0.92 |
| Cyclobacteriaceae | 0.09 | 0.00** | –1.14 | 0.00** | –0.65 | 010 | –0.65 | 0.07 | 0.07 | 0.85 |
| o__Thalassobaculales | –0.26 | 0.00** | 0.21 | 0.60 | 0.21 | 0.64 | 0.12 | 0.76 | 0.16 | 0.68 |
| o__Enterobacterales | –2.10 | 0.00** | –0.04 | 0.91 | –0.98 | 0.02** | 0.04 | 0.91 | 0.05 | 0.90 |
| Unknown family | 0.23 | 0.00** | –1.17 | 0.00** | 0.05 | 0.91 | –0.77 | 0.05 | 0.20 | 0.61 |

**Significant difference (*p*<0.05)

**Table S9. Raw data of the environmental variables of the seawater at both sampling locations.** Seawater samples were collected during two years at each climatic season (dry-2022, rainy-2022, dry-2023 and rainy-2023). Triplicate samples were analyzed for ammonia, phosphates, nitrates, nitrites, and total suspended solids (TSS), and temperature, salinity, pH and oxygen saturation and concentration were measured *in situ*. T: Temperature, S: Salinity, TSS: Total suspended solids.

| **Location** | **Year** | **Season** | **Date** | **R** | **Depth**  **(m)** | **T (°C)** | **S** | **pH** | **Sat. O_2_**  **(%)** | **O_2_**  **(mg L**^–^**^1^)** | **Ammonia**  **(µg N L**^–^**^1^)** | **Phosphates**  **(µg P-PO_4_ L**^–^**^1^)** | **Nitrates**  **(µg N-NO_3_ L**^–^**^1^)** | **Nitrites**  **(µg N-NO_2_ L**^–^**^1^)** | **TSS**  **(mg L**^–^**^1^)** |
| --- | --- | --- | --- | --- | --- | --- | --- | --- | --- | --- | --- | --- | --- | --- | --- |
| Protected | 2022 | Dry | 22-mar-22 | 1 | 9.20 | 26.40 | 36.10 | 8.91 | 72.00 | 5.62 | 10.00 | 2.00 | 2.10 | 0.91 | 3.69 |
|  |  |  |  | 2 | 11.34 | 26.20 | 36.30 | 8.18 | 67.60 | 6.01 | 10.00 | 2.00 | 5.58 | 1.46 | 3.61 |
|  |  |  |  | 3 | 8.46 | 26.50 | 36.20 | 8.63 | 79.50 | 6.30 | 11.60 | 2.00 | 7.25 | 1.58 | 3.57 |
|  |  | Rainy | 14-oct-22 | 1 | 9.20 | 28.20 | 29.90 | 8.40 | 54.90 | 4.29 | 10.00 | 4.70 | 12.50 | 0.70 | 12.20 |
|  |  |  |  | 2 | 11.34 | 28.30 | 29.40 | 8.43 | 56.90 | 4.22 | 10.00 | 3.32 | 11.90 | 0.70 | 11.10 |
|  |  |  |  | 3 | 8.46 | 28.30 | 29.90 | 8.45 | 57.10 | 4.30 | 10.00 | 3.26 | 11.10 | 0.70 | 11.00 |
|  | 2023 | Dry | 13-abr-23 | 1 | 9.20 | 26.00 | 35.70 | 8.12 | 73.50 | 5.90 | 11.40 | 4.64 | 5.16 | 1.56 | 15.90 |
|  |  |  |  | 2 | 11.34 | 26.40 | 35.29 | 8.13 | 73.50 | 6.07 | 10.00 | 3.28 | 6.60 | 1.35 | 13.00 |
|  |  |  |  | 3 | 8.46 | 25.84 | 35.70 | 8.12 | 73.50 | 5.90 | 10.00 | 3.13 | 5.27 | 1.39 | 14.20 |
|  |  | Rainy | 3-nov-23 | 1 | 9.20 | 30.00 | 29.30 | 8.60 | 109.80 | 6.05 | 10.00 | 2.00 | 32.60 | 0.70 | 3.93 |
|  |  |  |  | 2 | 11.34 | 30.20 | 30.00 | 8.60 | 95.30 | 6.92 | 13.30 | 2.00 | 17.70 | 0.70 | 4.01 |
|  |  |  |  | 3 | 8.46 | 30.40 | 30.40 | 8.60 | 72.50 | 5.62 | 10.00 | 2.00 | 10.80 | 0.96 | 4.25 |
| Urban | 2022 | Dry | 22-mar-22 | 1 | 3.96 | 25.90 | 36.30 | 8.35 | 65.00 | 5.39 | 11.60 | 3.28 | 21.70 | 1.63 | 1.40 |
|  |  |  |  | 2 | 3.96 | 26.60 | 36.10 | 8.34 | 81.70 | 5.70 | 11.80 | 3.09 | 20.05 | 1.43 | 1.77 |
|  |  |  |  | 3 | 5.43 | 26.40 | 36.40 | 8.39 | 63.00 | 5.22 | 13.40 | 2.83 | 21.60 | 1.56 | 1.62 |
|  |  | Rainy | 14-oct-22 | 1 | 3.96 | 28.40 | 29.80 | 8.30 | 57.10 | 4.16 | 10.00 | 4.50 | 9.78 | 0.70 | 23.10 |
|  |  |  |  | 2 | 3.96 | 28.70 | 29.90 | 8.43 | 55.90 | 4.44 | 10.00 | 4.53 | 13.80 | 0.70 | 14.30 |
|  |  |  |  | 3 | 5.43 | 28.20 | 29.70 | 8.45 | 58.30 | 4.30 | 10.00 | 4.57 | 9.99 | 0.70 | 34.20 |
|  | 2023 | Dry | 13-abr-23 | 1 | 3.96 | 26.40 | 35.60 | 8.01 | 83.10 | 6.71 | 10.00 | 2.56 | 2.10 | 0.70 | 15.00 |
|  |  |  |  | 2 | 3.96 | 26.40 | 35.40 | 8.17 | 86.00 | 6.79 | 10.00 | 3.91 | 2.10 | 0.70 | 14.70 |
|  |  |  |  | 3 | 5.43 | 26.20 | 35.30 | 8.22 | 88.60 | 7.26 | 10.00 | 2.00 | 2.10 | 0.70 | 27.60 |
|  |  | Rainy | 3-nov-23 | 1 | 3.96 | 30.20 | 31.20 | 8.56 | 130.20 | 8.20 | 10.00 | 2.00 | 13.20 | 0.70 | 3.39 |
|  |  |  |  | 2 | 3.96 | 30.20 | 31.20 | 8.72 | 94.40 | 8.25 | 10.00 | 2.00 | 2.14 | 0.70 | 3.30 |
|  |  |  |  | 3 | 5.43 | 30.10 | 30.70 | 8.70 | 101.70 | 7.56 | 10.00 | 2.00 | 3.10 | 0.70 | 3.28 |

**Table S10. PERMANOVA main test for the environmental data (physicochemical) of the seawater.** Factors ‘Site’ (levels: protected and urban) and ‘Season’ (levels: dry-2022, rainy-2022, dry-2023 and rainy-2023) were tested for differences. MS: mean square, P: permutations, U Perms: unique permutations, P (MC): *p*-value for Monte-Carlo post hoc test.

| **Environmental** | **Degrees of**  **freedom** | **Sum of squares** | **MS** | **Pseudo-*F*** | **P(Perm)** | **U Perms** | **P (MC)** |
| --- | --- | --- | --- | --- | --- | --- | --- |
| Site | 1 | 2.98 | 2.98 | 1.58 | 0.179 | 998 | 0.196 |
| Season | 3 | 142.26 | 47.42 | 25.18 | 0.001** | 998 | 0.001** |
| Site × Season | 3 | 31.63 | 10.54 | 5.59 | 0.001** | 996 | 0.001** |
| Residual | 16 | 30.13 | 1.88 |  |  |  |  |
| Total | 23 | 207 |  |  |  |  |  |

**Significant difference (*p*<0.05)

**Table S11. PERMANOVA pairwise test for the environmental data of the seawater.** Factor ‘Season’ (levels: dry-2022, rainy-2022, dry-2023 and rainy-2023) was tested for differences. Perm: permutations, U Perms: unique permutations, *p*-value (MC): *p*-value for Monte-Carlo post hoc test.

| **Groups** | ***t*** | ***p*-value (perm)** | **U Perms** | ***p*-value (MC)** |
| --- | --- | --- | --- | --- |
| Dry2022-Rainy2022 | 5.965 | 0.001** | 991 | 0.001** |
| Dry2022-Dry2022 | 3.9 | 0.001** | 990 | 0.001** |
| Dry2022-Rainy2023 | 3.968 | 0.001** | 988 | 0.001** |
| Rainy2022-Dry2023 | 6.768 | 0.001** | 988 | 0.001** |
| Rainy2022-Rainy2023 | 5.238 | 0.001** | 987 | 0.001** |
| Dry2023-Rainy2023 | 5.364 | 0.001** | 995 | 0.001** |

**Significant difference (*p*<0.05)

**Table S12. SIMPER test results for the environmental data of the seawater.** Similarity percentages and contribution of each variable using Euclidean distance. Sq.Dis/SD: average square distance or standard deviation. Cont.: contribution (%).

| **Group (Season)** | **Dry-2022** | | **Rainy-2022** | | **Dry-2023** | | **Rainy-2023** | |
| --- | --- | --- | --- | --- | --- | --- | --- | --- |
| **Variable** | **Sq. Dist/SD** | **Cont. (%)** | **Sq. Dist/SD** | **Cont. (%)** | **Sq. Dist/SD** | **Cont. (%)** | **Sq. Dist/SD** | **Cont. (%)** |
| Salinity | 0.58 | 0.03 | 0.49 | 0.74 | 0.62 | 0.12 | 0.55 | 1.79 |
| Temperature | 0.52 | 0.49 | 0.48 | 1.69 | 0.57 | 0.77 | 0.51 | 0.14 |
| pH | 0.53 | 24.79 | 0.47 | 8.87 | 0.50 | 3.28 | 0.58 | 1.91 |
| Oxygen | 0.56 | 2.08 | 0.52 | 1.37 | 0.59 | 5.72 | 0.59 | 15.13 |
| TSS | 0.63 | 3.09 | 0.55 | 42.1 | 0.45 | 3.36 | 0.61 | 0.31 |
| Ammonia | 0.55 | 27.82 | – | 0 | 0.44 | 10.71 | 0.44 | 39.54 |
| Nitrites | 0.46 | 8.25 | – | 0 | 0.63 | 38.80 | 0.44 | 2.57 |
| Nitrates | 0.57 | 24.54 | 0.57 | 3.89 | 0.63 | 10.92 | 0.58 | 38.6 |
| Phosphates | 0.63 | 8.91 | 0.59 | 41.34 | 0.55 | 26.32 | – | 0 |

**Table S13. Selection of the most representative variables for the canonical correspondence analysis (CCA).** The variance inflation detects the linear dependency of the variables, values <10 indicates non collinearity. CCA1 and CCA2 components, R^2^ value and the statistic test to detect significant variable in the ordination.

| **Variable** | **Variance inflation** | **Collinear** | **CCA1** | **CCA2** | **R^2^** | ***p*-value** |
| --- | --- | --- | --- | --- | --- | --- |
| Temperature | 30.77 | Yes | – | – | – | – |
| Salinity | 36.42 | Yes | – | – | – | – |
| pH | 2.55 | No | 0.86 | –0.51 | 0.11 | 0.055 |
| Oxygen | 6.25 | No | –0.99 | 0.11 | 0.25 | 0.001** |
| Ammonia | 1.89 | No | 0.54 | 0.83 | 0.52 | 0.001** |
| Phosphates | 3.06 | No | 0.99 | –0.02 | 0.01 | 0.651 |
| Nitrates | 2.21 | No | 0.84 | 0.53 | 0.96 | 0.001** |
| Nitrites | 4.32 | No | –0.20 | 0.98 | 0.55 | 0.001** |
| TSS | 3.38 | No | –0.07 | 0.99 | 0.57 | 0.001** |

**Significant difference (*p*<0.05)

**Table S14. ANOVA-like permutational test results.** Significance test for each variable in the canonical correspondence analysis (CCA). Df: degrees of freedom, CCA1: principal coordinate 1, CCA2: principal coordinate 2.

| **Variable** | **Df** | ***F*** | ***p-*value** | **CCA1** | **CCA2** |
| --- | --- | --- | --- | --- | --- |
| Oxygen | 1, 71 | 0.916 | 0.001** | –0.436 | 0.474 |
| Ammonia | 1, 71 | 0.468 | 0.001** | 0.574 | 0.538 |
| Nitrates | 1, 71 | 1.022 | 0.001** | 0.971 | 0.142 |
| Nitrites | 1, 71 | 1.136 | 0.001** | 0.120 | 0.836 |
| TSS | 1, 71 | 0.656 | 0.001** | –0.322 | –0.729 |

**Significant difference (*p*<0.05)
